# Enhanced Optical Spectroscopy for Multiplexed DNA and Protein-Sequencing with Plasmonic Nanopores: Challenges and Prospects

**DOI:** 10.1021/acs.analchem.1c04459

**Published:** 2022-01-01

**Authors:** Wang Li, Juan Zhou, Nicolò Maccaferri, Roman Krahne, Kang Wang, Denis Garoli

**Affiliations:** † State Key Laboratory of Analytical Chemistry for Life Science School of Chemistry and Chemical Engineering, Nanjing University, Nanjing 210023, P. R. China; ‡ Department of Physics and Materials Science, University of Luxembourg, L-1511 Luxembourg, Luxembourg; § Department of Physics, Umeå University, Linnaeus väg 20, SE-90736 Umeå, Sweden; ∥ Istituto Italiano di Tecnologia, Optoelectronics Research Line, Morego 30, I-16163 Genova, Italy

Plasmonics is the discipline that investigates the use of collective oscillations of conductive electrons in metallic nanostructures, called surface plasmons (SPs), to realize a large set of devices to be applied in sensing, nanomedicine, metamaterials, energy harvesting, and many others.^1^ During the past decade, several examples of plasmonic platforms have been proposed for single-molecule studies.^2−5^ Among others, plasmonic nanopores, i.e., sub-100 nm apertures connecting two compartments, are finding more and more interest as a specific family of solid-state nanopores with multiple functionalities. While the reader can find exhaustive details on working principles, fabrication, and applications of plasmonic nanopores for biosensing in recent reviews,^6,7^ here we focus on the applications of plasmonic nanopores as a platform for enhanced spectroscopy of single DNA and protein molecules, discussing in detail which limitations must be overcome to enable large scale multiplexing sequencing.

DNA single molecule sequencing is an established technology with two main approaches strongly present in the market, such as those by Pacific Biosciences^8^ and Oxford Nanopore Technologies.^9^ On the contrary, the effort needed to achieve single molecule protein sequencing still appears enormous, even if recent existing works have demonstrated that this idea could become a reality.^10−14^ The two major limitations in protein sequencing are the large number (20) of different amino acids to be discriminated and the unique nature of every polypeptide that cannot be replicated as in the case of DNA. The nanopore-sequencing technologies are nowadays the most intensively investigated single molecule sequencing approach. It is based on threading of single molecules of DNA or protein through a nanopore, where *k*-mers of polymer elements (*k* = 4–5) are detected as they are translocated through the pore in single element steps. Ion channels and bacterial toxin channels, e.g., α-hemolysin, MspA, and CsgG when embedded in a lipid bilayer membrane, serve as nanopore sensors with outstanding signal reproducibility and good sensitivity.^13,15^ Some engineered (i.e., mutated amino-acid sequence) versions of these channels have the right performance to allow DNA sequencing with single-pass accuracy close to 90%, and higher accuracy (up to 99.9%) can be achieved with multiple pass reads. Additionally, very recently, some proofs of concept of single protein sequencing using a biological nanopore with proper unfoldase to enable controlled polypeptide unfolding and translocation have been reported.^10,12,16^ Unfortunately, even with these outstanding advances, biological nanopores still present severe limitations, such as their bad stability and the complex procedures to perform their engineering. Moreover, their use in simultaneous multiplexed readout from many thousands of nanopores, without compromising the temporal bandwidth and sensitivity, is currently a huge challenge. In contrast, solid-state nanopores are actually extensively explored due to the potential of tuning both their physical and chemical properties.^17,18,27−36,19,37−40,20−26^ Sequencing by means of solid-state nanopores is not yet possible due to some major limitations such as time and space resolution and not an easy control of the translocation mechanism as in the case of biological nanopores with integrated molecular motors.^41−43^ In this context, plasmonic nanopores are the most interesting case of solid-state nanopores to realize complemented sensing with multiple measuring modalities beyond the mostly used electrical recording. Plasmonic nanopores offer improvements in sensitivity, specificity, observation rate, dwell time, and scalable parallelized detection. In a plasmonic nanopore, it is easy to confine and enhance the electromagnetic (EM) radiation to a nanoscale volume (hotspot), in which biomolecules can be “scanned” as they translocate through the pore with a significant improvement in signal-to-noise ratio due to enhanced optical detection.^7^ The implementation of an optical read-out scheme in a plasmonic nanopore enables one to perform measurements in the far-field, which provide a signal independent from the electrical one. Even more importantly, different techniques can be implemented within a nanopore sensor based on optical readout, such as single-molecule fluorescence,^44^ Forster resonance energy transfer (FRET),^45^ and surface enhanced Raman spectroscopy (SERS).^46^ In particular, fluorescence is extensively used in sequencing applications, but it presents a limitation that is impossible to be overcome related to the maximum number of colors that can be detected (up to six) without spectral overlap. This strongly limits multiplexing detection such as in the case of protein sequencing^12^ where up to 20 different signals should be decoded. On the contrary, both FRET and SERS enable additional multiplexing. Lifetime and intensity multiplexing are demonstrated methods to increase the number of color channels in FRET spectroscopy,^47,48^ and a number of different channels up to 10 is technologically feasible. On the contrary, SERS has less limitations in terms of multiplexing. In fact, every molecule has its own Raman fingerprint, and the use of SERS to discriminate among the 4 nucleotides and the 20 amino-acids has been reported and demonstrated.^49,50^ Another key advantage related to the optical readout is the intrinsic fast nature of the excitation/emission of photons, compatible with the short duration (typically below the microsecond range) of interaction between the single molecule and the nanopore. The fast translocation of the molecule through the pore makes the electrical measurements very noisy. However, if a fast optical detection can be implemented, the optical readout can be intrinsically less noisy. Thanks to plasmonics, the fast translocation of the molecule can be modified by exploiting some control mechanisms, such as optical and thermal forces, whose related phenomena are known as trapping and thermophoresis, respectively. These have shown great potential, and new methods in this field can significantly impact the commercial use of these technologies.

In this critical review, we discuss the current advantages and limitations of plasmonic nanopores as solid-state platforms for single molecule sequencing applications. We first highlight recent experiments on single-molecule detection in plasmonic platforms where the engineered EM field boosts the sensitivity and, at the same time, generates thermo-mechanical effects for controlling the molecule translocations through the nanopores. Furthermore, we discuss the three major optical readout techniques that can be implemented in a plasmonic nanopore (Figure 1), i.e., fluorescence, FRET, and SERS, and we will try to give some prospects on how optimization of these methods can facilitate real sequencing applications.

**Figure 1:**
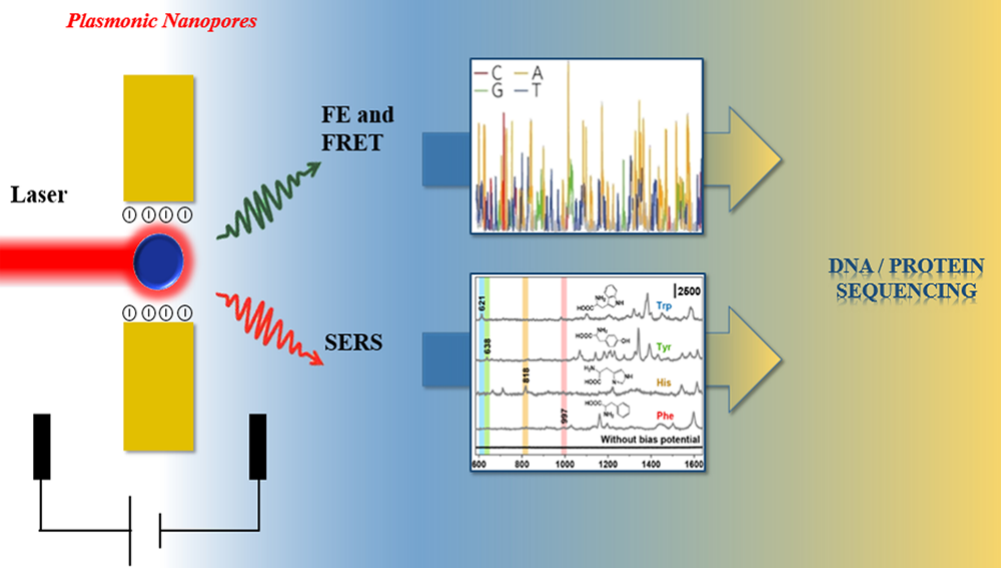
Schematic illustration of the principle of the plasmonic nanopore and its application of enhanced optical spectroscopy for multiplexed DNA and protein sequencing. Inset (bottom panel): reproduced from Yang, J. M.; Jin, L.; Pan, Z. Q.; Zhou, Y.; Liu, H. L.; Ji, L. N.; Xia, X. H.; Wang, K. *Anal. Chem.***2019**, *91* (9), 6275–6280 (ref (51)). Copyright 2019 American Chemical Society.

## Advanced Functionalities Offered by Plasmonic Nanopores

**Figure 2:**
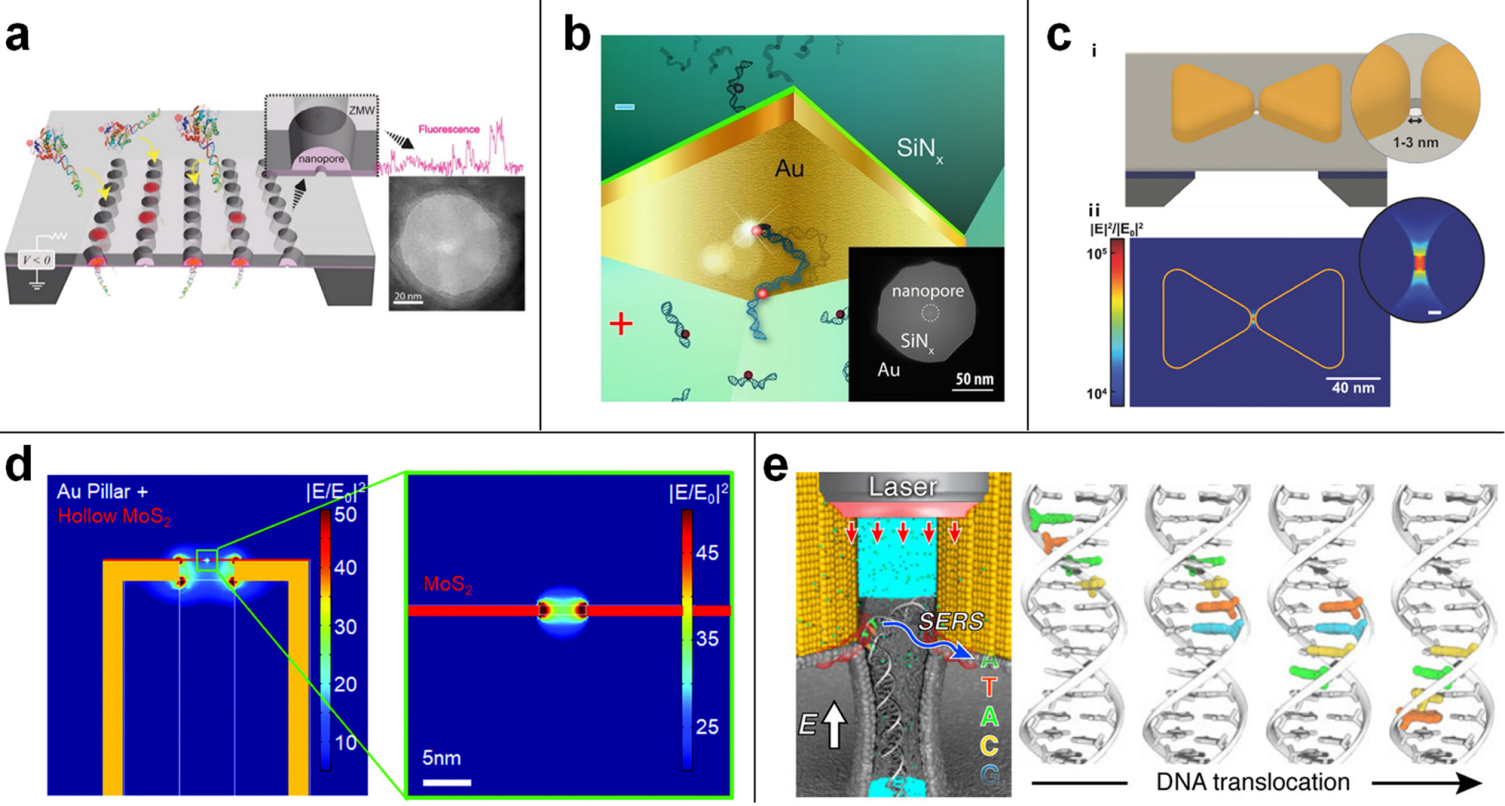
(a) SEM images of a nanopore zero-mode waveguides array and its setup for SMRT sequencing. Reproduced with permission from Larkin, J.; Foquet, M.; Turner, S. W.; Korlach, J.; Wanunu, M. Reversible Positioning of Single Molecule inside Zero-Mode Waveguide. *Nano Lett.***2014**, *14*, 6023–6029 (ref (61)). Copyright 2014 American Chemical Society. (b) Schematic illustration of the DNA translocation through a plasmonic nanopore, in which the ionic current flowing through the nanopore and the fluorescence emissions are probed in a synchronous manner. Reproduced from Light-Enhancing Plasmonic-Nanopore Biosensor for Superior Single-Molecule Detection, Assad, O. N.; Gilboa, T.; Spitzberg, J.; Juhasz, M.; Weinhold, E.; Meller, A. *Adv. Mater.* Vol. *29*, Issue 9 (ref (44)). Copyright 2017 Wiley. (c) Schematic of a solid-state nanopore with an integrated bowtie-antenna structure and the simulated electromagnetic field intensity. Reproduced from Integrating Sub-3 nm Plasmonic Gaps into Solid-State Nanopores, Shi, X.; Verschueren, D.; Pud, S.; Dekker, C. *Small*, Vol. *14*, Issue 18 (ref (64)). Copyright 2018 Wiley. (d) Electromagnetic field distribution with a disk with a MoS_2_ monolayer on top of the pillar. Reproduced from Garoli, D.; Mosconi, D.; Miele, E.; Maccaferri, N.; Ardini, M.; Giovannini, G.; Dipalo, M.; Agnoli, S.; De Angelis, F. *Nanoscale***2018**, *10* (36), 17105–17111 (ref (67)), with permission of The Royal Society of Chemistry. (e) Optical trapping and stepwise movement of DNA segment in a plasmonic nanopore. Reproduced from Belkin, M.; Chao, S.; Jonsson, M. P.; Dekker, C.; Aksimentiev, A. *ACS Nano***2015**, *9* (11), 10598–10611 (ref (68)). Copyright 2015 American Chemical Society.

The research and development works performed during the past decade have enabled single molecule DNA sequencing both on solid-state photonic platforms and on biological nanopore platforms.^52,53^ While in the most used solid-state platforms, the DNA sequencing is obtained by reading the nucleobase sequence via the duplication of ssDNA with fluorescent (four colors) complementary nucleic acids, in platforms based on nanopore, the sequencing is typically done by measuring current blockage during the translocation of single elongated molecule through the pore.^52−55^ A huge advancement in resolution is the major requirement to apply the current single-molecule methods to the new field of protein sequencing.^10^ A protein can be made of 20 different amino-acids, and distinguishing 20 different signal levels in optical or electrical read-outs is less than trivial. Combining electro-optical detection based on solid-state nanopores^56−59^ could be the way to improve the sensitivity and to enable low error-rate nanopore sequencing of both DNA and protein. Electro-optical single molecule detection has been recently discussed by other authors.^59^ This approach offers important advantages with respect to standard electrical read-out measurements performed in solid-state nanopores, but to date it has not been possible to demonstrate it in sequencing applications. Probably the unique example of solid-state nanopore sequencing is the recent paper reported by Wanunu and colleagues in collaboration with Pacific Bioscience on the use of a zero mode waveguide (ZMW) modified with nanopores to demonstrate the improved loading of long DNA molecule (Figure 2a).^60,61^ There, the sequencing has been possible thanks to the sequencing by synthesis of Pacific Bioscience (where a polymerase is bonded to the surface of the ZMW and used to duplicate the diffusing ssDNA molecule by four color fluorescent complementary nucleic acids).^52^

One of the key aspect related to plasmonic nanopores is the potential implementation of both electrical and enhanced optical read-out in the same platform. Several pioneer works have been reported on the use of plasmonic nanopores to detect single molecules during the translocation and compared the optical signals with the current blockade obtained during the simultaneous read-out. An example of electro-optical measurements performed by using a plasmonic nanopore has been reported by Assad et al. (Figure 2b).^44^ This simple plasmonic platform demonstrated to offer a high signal-to-background ratio for single-molecule detection at low excitation laser intensity while maintaining an extremely high temporal bandwidth for single-DNA sensing. The detection of photons emitted from the fluorescent tags linked to the DNA molecule and of current blockade obtained during the translocation demonstrated the complementary nature of this methodology. Many other examples of simultaneous electro-optical detection by means of a plasmonic nanopore have been reported.^44,56,62,63^ In particular, the most explored platform used paired nanoantennas to generate strong EM field enhancement at the nanogap where the nanopore can be prepared with a diameter comparable with that of typical biological pores (i.e., <3 nm) (Figure 2c).^64^ Different configurations have been proposed and demonstrated for single-molecule detection eventually combining electrical and optical read-outs: nanorods,^63^ bow-tie,^64^ inverted bow-tie,^65^ rectangular apertures,^66^ and paired-nanoholes.^6^

The rationale behind the investigation of several metallic nanostructure designs is to optimize the EM confinement and enhancement at the nanopore interface. This is the main parameter to obtain a gain in photon excitation and/or emission processes, such as fluorescence, FRET, and SERS being the major spectroscopic techniques used in plasmonic nanopores. The optimization of the EM field confinement is a key aspect related to the properties of space dependency of the signal intensity in optical spectroscopies. For example, SERS is effective within about 1 nm from the plasmonic nanostructure,^69^ and FRET is sensitive to the distance between donor and acceptor dyes with nanometer resolution.^47,48,70^ Therefore, in a plasmonic nanopore, biomolecules like DNA and protein produce strong FRET emission or SERS spectra only from the segments in the hot spot. Other parts not in close proximity to the hot spot can hardly give comparable signal, which makes optical read-out highly competitive with the electrical method once high spatial resolution in EM field confinement can be achieved.^71^ With this aim, high spatial resolution can be obtained by improving the design and fabrication process of the plasmonic nanopores, in particular the integration with 2D materials, such as MoS_2_ and others,^67,72,73^ which enables confining the EM field in a very narrow volume comparable with the thickness of the 2D layer (<1 nm) (Figure 2d). Worthy of being mentioned, solid-state nanopores prepared in 2D materials have been proved to be a potentially valuable solution toward protein and DNA sequencing.^74^

**Figure 3:**
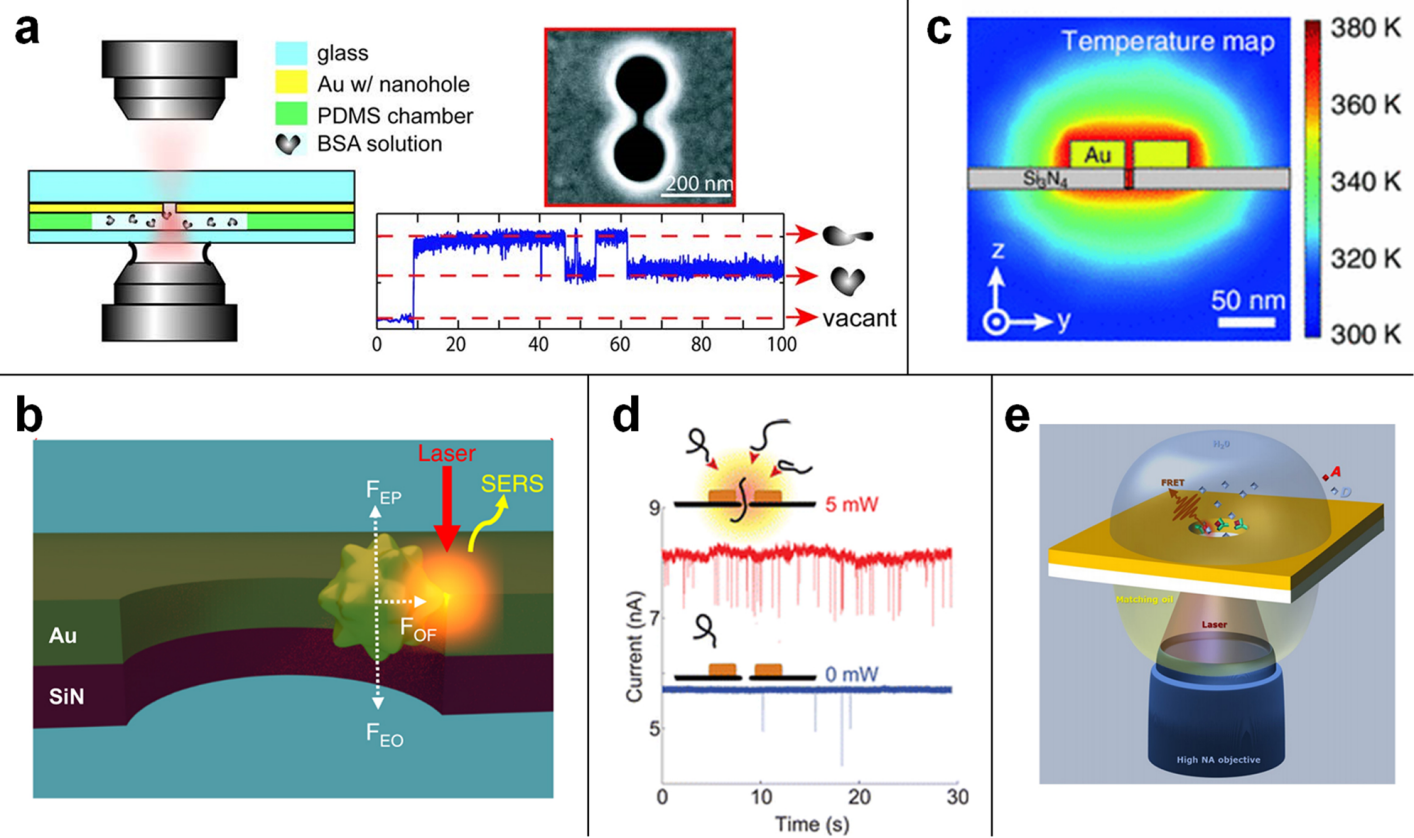
(a) Optical trapping of a single protein using a double-nanohole in an Au film. Reproduced from Pang, Y.; Gordon, R. *Nano Lett.***2012**, *12*, 402–406 (ref (76)). Copyright 2012 American Chemical Society. (b) The trapping due to a balance between the electrophoretic (FEP), electroosmotic (FEO), and optical (FOF) forces leads to a plasmonic hot spot between the Au nanostar tip and the nanohole sidewall that allows single-molecule SERS. Reprinted by permission from Macmillan Publishers Ltd.: NATURE, Huang, J.; Mousavi, M. Z.; Zhao, Y.; Hubarevich, A.; Omeis, F.; Giovannini, G.; Schütte, M.; Garoli, D.; De Angelis, F.*Nat. Commun.***2019**, *10* (1), 5321 (ref (50)). Copyright 2019 under a Creative Commons Attribution 4.0 International License (http://creativecommons.org/licenses/by/4.0/). (c) The temperature map of a plasmonic nanopore. Reproduced with permission from Belkin, M.; Chao, S.-H.; Giannetti, G.; Aksimentiev, A. Modeling Thermophoretic Effects in Solid-State Nanopores. *J. Comput. Electron.*, **2014**, *13* (4), 826–838 (ref (21)). Copyright 2014 Springer Nature. (d) Schematic illustration of a DNA molecule translocating through a plasmonic nanopore that consists of a gold bowtie antenna with a 10 nm nanopore at the gap center and the event rate enhancement by localized plasmonic heating. Reproduced from Nicoli, F.; Verschueren, D.; Klein, M.; Dekker, C.; Jonsson, M. P. *Nano Lett.***2014**, *14*, 6917–6925 (ref (88)). Copyright 2014 American Chemical Society. (e) Schematic illustration of the plasmonic nanopore configuration for FRET between donor (D) and acceptor (A). Reprinted figure with permission from Zambrana-Puyalto, X.; Ponzellini, P.; Maccaferri, N.; Garoli, D. Förster-Resonance Energy Transfer between Diffusing Molecules and a Functionalized Plasmonic Nanopore. *Phys. Rev. Appl.***2020**, *14* (5), 054065 (ref (45)). Copyright 2020 by the American Physical Society.

The spatial resolution is not the only important limitation to be overcome in solid-state nanopores. One of the major differences between solid-state and biological nanopores is the control over molecular translocation. While molecular motors in biological nanopores have been proved to be a powerful tool, in solid-state nanopores, the control of translocation speed is still a major challenge. In this framework, plasmonic nanopores have been recently explored as a multifunctional platform to enable not only enhanced optical spectroscopy but also translocation control. Two main phenomena can be exploited and engineered in plasmonic nanopores, i.e., optical tweezing/trapping and localized optical heating.^75−77^ Plasmonic nanostructures have been extensively used to trap and control the dynamics of molecules and particles with sizes down to 10 nm. Nanostructures like nanopillars,^77^ double nanopillars,^78^ double nanopyramids,^78^ and coaxial and double nanohole apertures^79^ have been reported, and the reader can find an updated review on the topic in Kolbow et al.^80^ In the field of plasmonic nanopores, the pioneering paper of Belkin et al.^68^ demonstrated that it could be theoretically possible to control the steplike translocation of a polynucleotide molecule (Figure 2e) thanks to the highly localized field at the tip of a gold nanoantenna, and now the fabrication of nanoantennas with a narrow gap in correspondence to the solid-state nanopore is an established procedure.^64,81^ Different designs ensure extremely localized EM field intensity (|*E*|^2^) enhancements (up to 10^4^) and the confinement of such an optical field can be engineered in order to fit with a single molecule. Simulations showed that a controlled translocation is possible, but the duration of interaction between the fragments of the molecule (i.e., k-mer of nucleic acids) is on the order of tens of nanoseconds, and up to now it has not been possible to demonstrate this approach in a real experiment. Moreover, a single polypeptide or polynucleotide is still very challenging to be trapped in a solid-state nanopore. On the contrary, trapping of larger entities, such as whole proteins or nanoparticles, has been reported also for plasmonic nanopores.^79^ In particular, two major platform configurations have been used during the recent years, i.e., double pore and metallic nanoparticles trapped in relatively large (>50 nm) pores. In the double pore configuration, the very narrow gap between two adjacent pores in a metallic film enables one to produce a huge EM field confinement, and the gradient of the EM field can stably trap single molecules or nanoparticles (Figure 3a).^82,79,76,77^ The second configuration, where a metallic nanoparticle is trapped inside a nanoslit or a nanopore has been reported in several papers.^83,46^ Most recently, it has been demonstrated that the use of a gold nanostar can enable the trapping of the particle up to minutes thanks to multiple effects, such as optical forces and electrodynamic effects.^49,50^ The significant advantage in using a nanostar is related to the sharp protrusion with a radius of curvature of about 1 nm. The forces generated in the system can push this “nanotips” in close contact to the nanopore (Figure 3b), hence generating a nanogap with a volume comparable with a nanocavity,^4^ i.e., close to 1 nm^3^. In this extremely narrow region, it can be possible to perform enhanced spectroscopy with unprecedented spatial resolution. Experimental results demonstrated that this platform enables nucleotide resolution in DNA analysis, while single amino-acid resolution could be achieved in spectroscopic investigation of short peptides.^49,50^ Unfortunately, this approach can be very useful only for single-molecule detection and analysis, while it can be hardly translated to real sequencing applications. More research is needed to make plasmonic nanopore trapping the tool to control molecular translocation in a comparable way as in biological nanopores.

Hence, optical tweezing is one of the explored approaches to reduce the DNA molecule translocation speed through nanopores. The translocation time of a polymer chain is dependent on the free-energy landscape and diffusion coefficient of the polymer chain.^84^ The tuning of the translocation time can be done by playing with the free-energy landscape and diffusion coefficient. As discussed by Luo et al.,^84^ the free-energy depends on many factors, such as the solvent properties, driving force, pore size, and others. Several parameters have been investigated both in biological and solid-state nanopores. For example, the solvent used and the value of the pH can significantly impact the translocation velocity.^85^ The translocation time can also be tuned by acting on the interaction between the biomolecule and the nanopore, for instance, by coating the nanopore surface with a specific functional coating.^86,87^ These and other methods can be also used in plasmonic nanopores, but they do not use the enhanced functionality provided by the metallic nanostructure. On the contrary, one of the most interesting functionalities offered by plasmonic nanopores is the local heat generation (Figure 3c).^21^ The translocation time also depends on the temperature of the system, and this could be used to act on the biomolecule diffusion. Both the conformational entropy and the diffusion coefficient of a polynucleotide or a polypeptide are dependent on the temperature. In particular, in solid-state nanopores, it has been demonstrated that the translocation time can be decreased by increasing the temperature.^88,23^ A localized optical heating produced by a plasmonic nanopore^89^ can induce a temperature gradient with the consequent increment of electrolyte conductivity and thermal diffusion of ions, the so-called thermophoresis effect.^21,90^ By using this approach, it is possible to enhance the ion current and modify the molecular capture rates (Figure 3d). The nanopore temperature control can be used for studying single-molecule thermal kinetics with submicrosecond time scale resolution but also to modify the capturing of a target molecule, which can be measured in order to obtain information from its sequence and so on.

## Toward Sequencing by Using Fluorescence Spectroscopy in Plasmonic Nanopores

Historically, DNA sequencing had been built on chemical methods and fluorescence spectroscopy of the four nucleotide letters to be decoded from the genome. Sanger sequencing and Illumina next generation sequencing are both based on four colors discrimination and demonstrated to be very precise and powerful methods to obtain the sequence from DNA or RNA.^55^ The use of fluorescence in single-molecule sequencing was first demonstrated in the ZMW platform, which later became the starting point of the Pacific Bioscience technology.^52^ Also in this case, a four-colors code is used to read the sequence from a ssDNA in a sequencing by synthesis method. Being a spectroscopic technique, single-molecule sequencing based on fluorescence has been extensively explored by using optimized plasmonic platforms. In particular, ZMW prepared with different geometries and materials improved the fluorescence intensities by a factor between a few tens up to 10^4^.^91−96^ Although these results were significant for single-molecule fluorescence experiments, they can hardly be translated to nanopore sequencing where no real-time synthesis of DNA is typically performed. As in other plasmonic platforms, in a plasmonic nanopore it is relatively easy to engineer the EM field in order to obtain high fluorescence enhancement at specific wavelengths. It has been demonstrated that sequential fluorescence signals can be detected during DNA translocation. To date, single-molecule detection has been performed in plasmonic nanopores by modifying one or two of the nucleobases with a fluorescent dye.^44^ This enabled to detect the molecule and to explore multiple optimization methods in nanopore design.^7^ Moreover, the use of one of two dyes linked to a specific position along a biomolecule can be used to investigate chemical modifications also at single residue resolution, an application that is now extensively investigated in solid-state nanopores by using an electrical read-out scheme.^97−99,57^ In order to move from single-molecule detection to sequencing, a four color discrimination should be implemented. Unfortunately, although it can be technically possible to decorate the different nucleotides with different dyes, to date, a DNA strand fully decorated with fluorescent modified nucleotides has not been reported. If such a modification to a single DNA molecule could be done, a plasmonic nanopore with a high enough EM field confinement combined with a very fast detector could be able to perform real-time single-molecule sequencing. Anyway, there are several major challenges to be overcome to achieve this goal. First of all, as previously mentioned, a control on the translocation velocity should be introduced. In a solid-state nanopore, a biomolecule interacts with the pore in a time scale of microseconds, making it extremely challenging to collect spectroscopic signals from thousands of sequential elements. Second, a typical geometry used in plasmonic nanopore technology^7^ enables one to confine the electromagnetic field in a volume comparable to tens of nucleotides (i.e., >10 nm^3^). Consequently the fluorescence enhancement is not limited to a single nucleotide, but multiples of them will be detected simultaneously. Third, the need to use fully decorated molecules can be in principle applied to DNA and RNA, but it is close to impossible for proteins where 20 different spectrally separated colors (dyes) simply do not exist.

While the use of 2D materials integrated with plasmonic nanopores demonstrated to enable field confinement down to 1 nm (comparable with the 2D material thickness),^67^ the detection of different colors emitted at a high rate (hundreds of kHz, considering an interaction between the dyes and the nanopore around 1 μs) is yet to be demonstrated. For these reasons, alternative approaches have been explored in order to use fluorescence for DNA and protein sequencing both in biological and solid-state nanopores.

In the search of novel methods for the use of fluorescence in single-molecule sequencing, the nanoscale energy transfer between fluorescent molecules, i.e., FRET, has been recently explored. When the distance between an excited donor molecule (D) to the ground-state acceptor molecule (A) is in the range of 1–20 nm, the energy transfer is described by formalism, which accounts for a near-field nonradiative dipole–dipole interaction. FRET is extensively used in multiple bioassays,^70^ while its use in single-molecule sequencing has been reported in some recent seminal works. In particular, Van Ginkel et al.^100^ used FRET in a first experimental demonstration of a single-molecule protein fingerprint. FRET has also been explored in plasmonic nanocavities and in nanopores. Zambrana-Puyalto et al. have recently reported on the enhancement of FRET efficiency in gold nanopores,^101^ while the same authors demonstrated how FRET can be used in a plasmonic nanopore to detect single dyes translocation (Figure 3e).^45^ As discussed in ref (7), the FRET efficiency is related to the probability of an energy-transfer event occurring per donor excitation event. As well, it depends on (i) the radiative decay rates of the donor and the acceptor, and (ii) on the rates of any other de-excitation pathways excluding energy transfers to other acceptors. Beyond the distance between the donor and the acceptor, the FRET efficiency depends on many factors, such as (i) the overlap between the donor emission and the acceptor absorption spectra and (ii) the relative orientation of the donor–acceptor dipole momenta. In this framework, plasmonics can play an additional role in enhancing the FRET by increasing the decay rate of D, A, or both.

More interesting, FRET phenomena enables a significant multiplexing in the fluorescence based read-out. In fact, depending on the distance between D and A and on the other parameters involved in FRET efficiency, the intensity and lifetime of the emission can be extensively modulated.^47,70,102,103^ This means that a single color can be in principle used to discriminate between different entities (such as different amino acids or nucleotides) interacting with a nanopore. FRET multiplexing has been recently proposed and theoretically demonstrated to be able to achieve 9 amino-acid discrimination in a protein translocating through a nanopore chemically functionalized with multiple couples of D and A.^104^ The label free peptide that translocates through the pore induces slight changes in the distance between the D/A couples depending on the different amino-acid inside the pore at a specific moment. In this way, the FRET emission resulted to be dependent on the single amino-acid with a high level of fidelity in the recognition. Although this method can be challenging to be implemented, it demonstrates how FRET represents a plus with respect to the standard few colors fluorescence spectroscopy in single-molecule sequencing development.

## Toward Sequencing by Using SERS Spectroscopy in Plasmonic Nanopores

**Figure 4:**
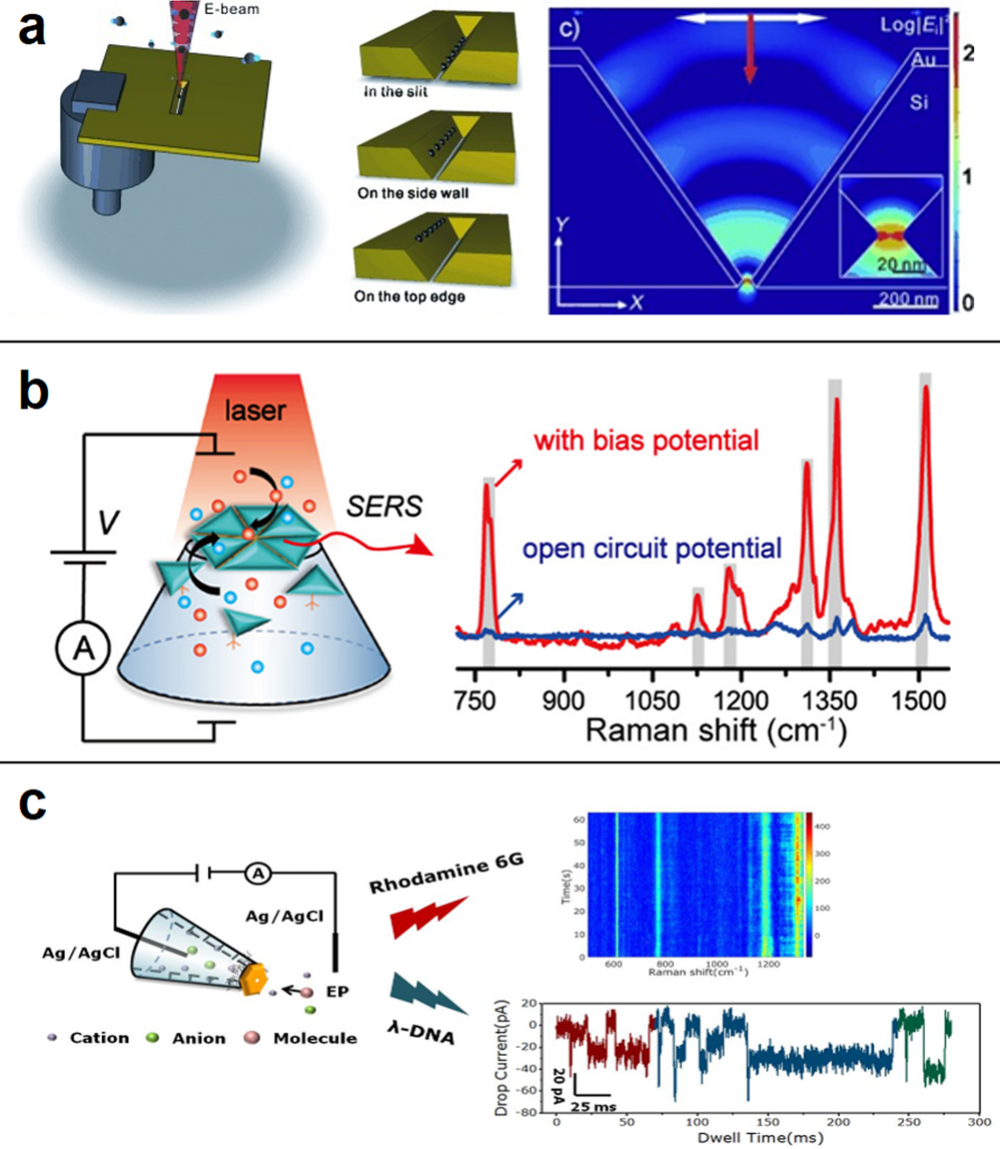
(a) Schematic illustration of the plasmonic nanoslit structure and its FDTD-simulated optical field enhancement distribution. Reprinted from Direct Evidence of High Spatial Localization of Hot Spots in Surface-Enhanced Raman Scattering. Chen, C.; Hutchison, J. A.; Clemente, F.; Kox, R.; Uji-I, H.; Hofkens, J.; Lagae, L.; Maes, G.; Borghs, G.; Van Dorpe, P. *Angew. Chem. Int. Ed.* Vol. *48*, Issue 52 (ref (113)). Copyright 2009 Wiley. (b) Schematic illustration of a silver nanotriangle-based nanopore system for detecting molecule translocation using surface-enhanced Raman scattering. Reproduced from Cao, J.; Liu, H. L.; Yang, J. M.; Li, Z. Q.; Yang, D. R.; Ji, L. N.; Wang, K.; Xia, X. H. *ACS Sens.***2020**, *5*, 7, 2198–2204 (ref (114)). Copyright 2020 American Chemical Society. (c) SERS and ionic current measurement in an Au nanoplate assembled glass nanopipet. Reprinted from *J. Electroanal. Chem.* Vol. *894*, Shen, Q.; Zhou, P. L.; Huang, B. T.; Zhou, J.; Liu, H. L.; Ahmed, S. A.; Ding, X. L.; Li, J.; Zhai, Y. M.; Wang, K. Mass transport through a sub-10 nm single gold nanopore: SERS and ionic current measurement, pp 115373 (ref (115)). Copyright 2021, with permission from Elsevier.

SERS contains the information on the characteristic vibrations of molecules, thereby enabling unambiguous identification of most biomolecules without the need for specific labeling. In addition to distinguishing 4 DNA bases and 20 amino acids,^105,106^ the SERS spectrum could provide comprehensive information on molecules including oxidation state, methylation, phosphorylation, and deprotonation.^107−110^ Combination of SERS read-out with plasmonic nanopore were first performed on silicon nanoslit–cavity fabricated by MEMS (microelectromechanical systems). In this case, the hot spot can be spatially localized at the center of the nanoslit (Figure 4a).^111^ Moreover, in order to further enhance the EM field intensity in the hot spot, Bragg mirror grooves can be arranged on both sides of the nanoslit.^112^ This improved configuration enabled one to achieve subnanometer spatial resolution and single-molecule SERS detection of four nucleobase adsorbed inside nanoslits.^66^ These pioneering works demonstrated that single-molecule SERS sensitivity could be reached by a rational engineering of the nanopore configuration. However, due to stochastic adsorption of the molecule into the nanocavity, it seems not possible to achieve sequential reads of the DNA strand or protein. Therefore, extra strategies to enable controllable translocation are needed for plasmonic nanopore sequencing.

**Figure 5:**
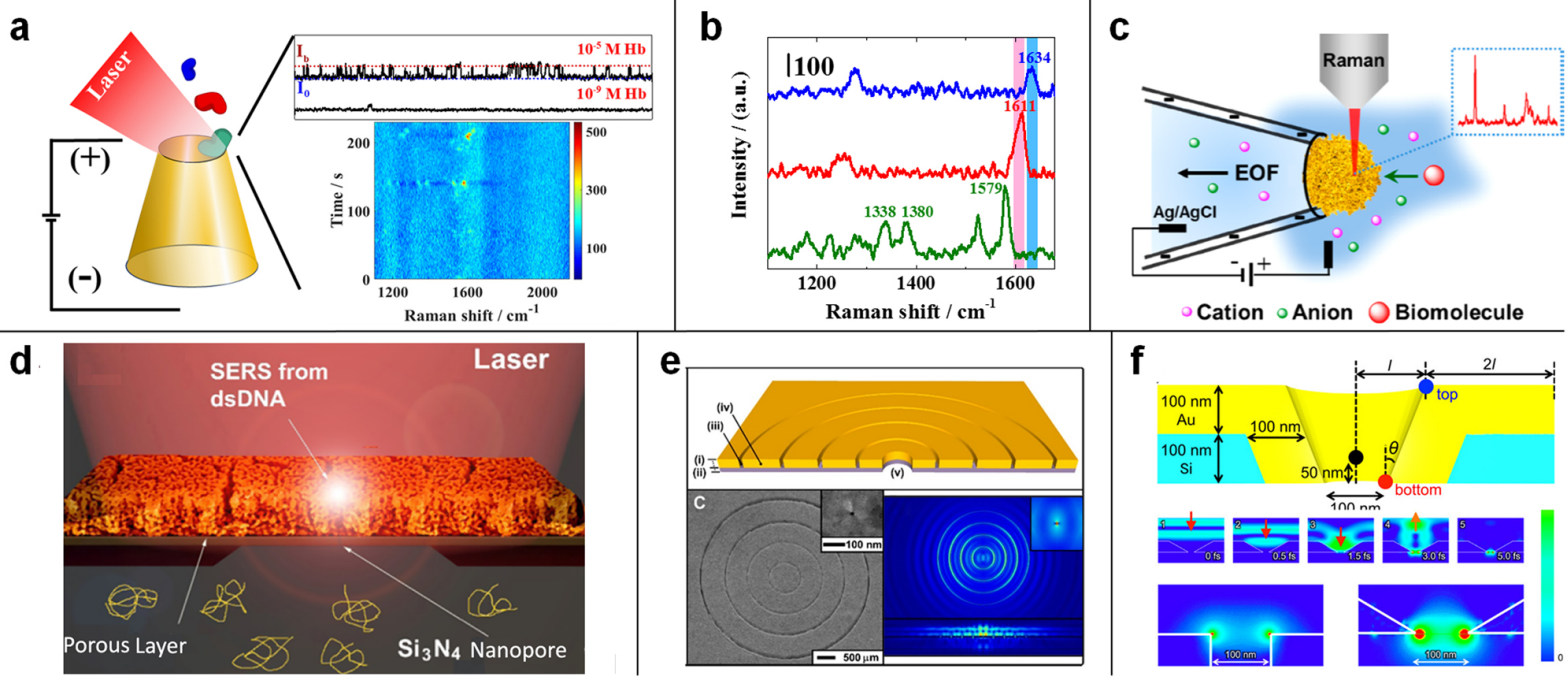
(a,b) Schematic graph of single proteins detected by SERS when transporting the gold conical nanopore. The shift in SERS spectra indicates the oxy- and deoxy-state of proteins. Reproduced from Zhou, J.; Zhou, P. L.; Shen, Q.; Ahmed, S. A.; Pan, X. T.; Liu, H. L.; Ding, X. L.; Li, J.; Wang, K.; Xia, X. H. *Anal. Chem.***2021**, *93* (34), 11679–11685 (ref (117)). Copyright 2021 American Chemical Society. (c) Nanoporous gold nanoparticle assembled on the nanopipet for SERS detection under a bias potential. Reproduced from Yang, J. M.; Jin, L.; Pan, Z. Q.; Zhou, Y.; Liu, H. L.; Ji, L. N.; Xia, X. H.; Wang, K. *Anal. Chem.***2019**, *91* (9), 6275–6280 (ref (51)). Copyright 2019 American Chemical Society. (d) A plasmonic nanopore prepared in a thick nanoporous film supported by a Si_3_N_4_ membrane. Reproduced from Hubarevich, A.; Huang, J. A.; Giovannini, G.; Schirato, A.; Zhao, Y.; Maccaferri, N.; De Angelis, F.; Alabastri, A.; Garoli, D. *J. Phys. Chem. C***2020**, *124* (41), 22663–22670 (ref (119)). Copyright 2020 American Chemical Society. (e) A plasmonic nanopore surrounded by bullseye structure with a localized heating spot inside the nanopore. Reproduced from Crick, C. R.; Albella, P.; Kim, H. J.; Ivanov, A. P.; Kim, K. B.; Maier, S. A.; Edel, J. B. *ACS Photonics***2017**, *4*, 11, 2835–2842 (ref (120)). Copyright 2017 American Chemical Society. (f) Side views of the cone-shaped plasmonic nanopore and the FDTD simulation results showed that the enhanced e.m. field was at the bottom and much stronger than conventional cylindrical plasmonic. Reprinted from Matsuda, R.; Ryuzaki, S.; Okamoto, K.; Arima, Y.; Tsutsui, M.; Taniguchi, M.; Tamada, K. Finite-Difference Time-Domain Simulations of Inverted Cone-Shaped Plasmonic Nanopore Structures. *J. Appl. Phys.***2020**, *127* (24), 243109 (ref (121)), with the permission of AIP Publishing.

As discussed in the previous sections, recent works theoretically predicted the utilization of optical gradient forces to achieve stepwise movement of DNA segments in a plasmonic nanopore.^68^ Moreover, it has been experimentally demonstrated that optical and electrophoretic forces can be combined to stably trap nano-objects inside a nanopore and to perform single-molecule SERS there with extreme spatial resolution.^49,50^ Anyway, to date, the experimental demonstration of SERS sequencing has not been possible. In order to achieve sequential SERS readout, the biomolecule should translocate through a small plasmonic nanopore, and the duration of the interaction between the molecule and hot-spot should be large enough to ensure a high signal-to-noise ratio. Two technical routes have been practiced to fabricate small plasmonic nanopores. The first route is direct fabrication of a single small plasmonic nanopore. Nanopores drilled in a Si_3_N_4_/Au membrane by a focused ionic beam have been frequently reported,^116,88^ but only a few studies employed SERS detection,^46^ probably because the pore size is still large (mostly larger than 20 nm) and the used plasmonic pore configurations enable weak Raman enhancement. Conversely, a glass/quartz nanopipet decorated with plasmonic metal has provided a much smaller pore size and higher SERS activity. Wang’s group fabricated 30 nm and sub-10 nm nanopores by assembling Ag nanotriangles^104^ and Au nanoplates^105^ at the orifice of the nanopipets, respectively (Figure 4b,c). The nanopore allows detecting DNA bases during direct translocation by SERS. Further development of a cone-shaped Au nanopore with sub-10 nm pore size showed that the orientation of single Rhodamine 6G and oxygenated/deoxygenated states of single hemoglobin during translocation can be observed by SERS (Figure 5a,b).^117^

The second technical route is to combine the single nanopore with nanoporous plasmonic structure (Figure 5c,d).^118,119^ With application of the bias potential, molecules can pass through the nanoporous plasmonic structure and provide a strong SERS signal.^51^ Both technical routes can provide single molecule SERS sensitivity, but several challenges are still there. More importantly, the fabrication of plasmonic nanopores with controlled and reproducible pore sizes is still difficult. Although chemical synthesis of pores containing gold nanoplates with controllable pore size has been reported, manipulation and assembly of these nanoplates to form nanopore devices with good performance as single molecule SERS sensors still need a lot of effort.^115^

Second, different orientations of a single molecule inside the nanopore provides multiple chemical information on the biomolecule and complicates the analysis of sequencing as well. Various orientations of molecules usually cause the fluctuation of SERS spectra, causing peak shifts, appearances and disappearances, or intensity changes. Therefore, the mixed SERS signal need to be decoded by comprehensive algorithms.

Several common problems should also be solved before meaningful SERS-nanopore sequencing signal could be observed. First, slowing down the translocation speed of molecules and improving temporal resolution of the spectra collection are two important prerequisites to achieve plasmonic nanopore-based SERS sequencing. Typical translocation of a DNA base or an amino acid residual through solid-state nanopores takes nano- to microseconds, which is too fast compared with the millisecond per frame in SERS spectra acquisition.^109^ Improving temporal resolution of spectra collection is a key issue. Ultrafast Raman spectrometer equipped with a nanosecond pulse laser could be a good choice. Optical trapping, controlled steplike movement, and optical spectroscopy by plasmonic field pulses have been theoretically demonstrated to be feasible in SERS sequencing of DNA.^70^ Therefore, developing Raman spectrometer qualified for plasmonic nanopore sequencing is promising and feasible.

Except for collecting spectra at high speed, improving the detection sensitivity of SERS to a submolecular level is also fundamental for DNA and protein sequencing. Although present SERS nanopores have reached a single-molecule sensitivity, significant improvements are required to ensure precise spectroscopic differentiation of different segments in biomolecules. Three aspects need to be considered for improving the detection sensitivity. The first aspect is to further improve the Raman signal enhancement in the plasmonic nanopore. Both nanostructure configuration and the material used would influence the intensity and distribution of the electromagnetic field. Well-designed structures have been proved to greatly enhance the local electromagnetic field intensity inside the nanopore. For example, a plasmonic nanopore showed a higher enhancement factor in the presence of a surrounding bullseye structure (Figure 5e).^120^ The SERS enhancement factor in a gold bowtie^88^ or cone-shaped plasmonic nanopore^121^ (Figure 5f) was 1000 times stronger than that of a conventional cylindrical one. Besides, selection of the plasmonic metal for nanopore fabrication is also critical for obtaining a superhigh local EM field. Silver nanostructures usually show a stronger enhancement than gold, and research has found that DNA bases can be identified using a silver nanopore.^114^ Therefore, the silver nanopore is a good alternative if it can be protected from oxidation. Another important aspect to consider for SERS-nanopore sequencing regards the molecules adsorbed on the interface around the plasmonic nanopore. These no-translocating molecules can be the source of interference signals. Interference signals caused by the adsorbed biomolecule outside the plasmonic nanopore can be reduced by decreasing the total area of the plasmonic nanopore exposed to the objective focus. In light of this consideration, conical/pyramidal plasmonic nanopores with locally modified metal nanopore structure (instead of bulk sputtering) would be preferable. Besides, covering inert inorganic thin layer of SiO_2_, Al_2_O_3_^122^ using atomic layer deposition technique or adsorbing a monolayer of halide ions^123^ on the plasmonic nanopore may also hinder unwanted adsorption.

Finally, the combination of standard SERS with nonlinear Raman scattering-based techniques, such as surface enhanced hyperRaman scattering (SERRS)^124^ and surface enhanced anti-Stokes Raman scattering,^125^ can provide complementary spectroscopic information because these process obey different surface selection rules.^125,126^

## Outlook and Conclusions

The application of plasmonic platforms for sensing down to single-molecule sensitivity is an active research field that enables more and more advances toward different applications. Plasmonic nanopores, as members of the large family of solid-state nanopores, are now investigated to improve the functionality offered by semiconductor-based nanopores. The engineering of the electromagnetic field confinements and the consequent effects, such as localized optical forces, thermal effects, and enhanced spectroscopies make it possible to detect and control single molecules in a volume close to 1 nm^3^. As underlined in this paper, the key aspect in the use of plasmonic nanopores is the possibility to integrate high-sensitivity optical sensing with electrical read-out schemes. As previously discussed,^7^ this approach can be used in several experiments at single-molecule levels, with the huge advantage of applying multiple methods such as fluorescence, FRET, and SERS to further enhance the amount of information that comes from a single-molecule experiment. In this review, we tried to discuss the major limitations in the development of sequencing of DNA and protein by means of plasmonic nanopore platforms. Besides the additional functionalities that can enable one to a better control the single molecule translocation velocity, the goal of a full control of linearized DNA and protein molecules movement appears to be far achieved. The combination of optical, thermal, and electro-osmotic forces can help in this direction as they demonstrated a good control in nanoparticles translocation.^50^ Alternative methods could be explored, for example, the use of magnetic fields^127,128^ or the functionalization of the nanopore with functional coatings/molecules.^86,129^ Another approach to overcome the not-controlled translocation could be to investigate integrated designs, where the plasmonic solid-state pore is finalized with a biological pore so realizing a hybrid structure. It has been already demonstrated that a biological nanopore can be aligned and integrated into a solid-state pore,^33,130^ also in the case of a plasmonic platform.^131,7^ The obvious advantage of this very challenging to fabricate system is the combination of the functionality offered by the biological pore, such as the function of a molecular motor, and the enhanced field generated by the plasmonic nanostructures that can be, for example, used to facilitate the unfolding and capture in protein sequencing experiments. The second important issue that has been discussed is in regards to the optimization of the field confinement. With respect to biological nanopores where the pore thickness is typically on the order of 1 or 2 nm,^13,15^ solid-state nanopores are really challenging to have a thickness below 5 nm. This is also true for plasmonic nanopores, where metallic nanostructures need to be included in the design. The solution could be to use 2D materials integrated with plasmonic pores. In this case, the field can be confined down to a thickness comparable to the atomic thickness of the 2D material used, with a consequent huge improvement in spatial resolution.^67^ The final and most important issue regards the choice of the spectroscopic technology to be used to decode the complex information inside a DNA or a protein molecule. While fluorescence have been the most explored technique, it is clear that the major limitation in the small number of different colors that can be discriminated (without spectral overlap) in the visible range cannot be overcome. On the contrary, FRET multiplexing could enable one to read more signals even if the data analysis in terms of lifetime and intensity could be nontrivial. We believe that the most suitable spectroscopic technique could be SERS. Being a label free method with ease of multiplexing, SERS could be able to discriminate also among 20 amino-acids in a single protein. For example, by using an approach similar to the one reported in ref (49), it could be possible to obtain significant information about a protein’s amino-acid sequence by measuring, sequentially, the subfraction of the whole protein separated by means of specific peptidases. In this way, the few amino-acid resolution of the plasmonic nanopore can be used to reconstruct the whole sequence, even if a relatively large number of experiments are needed. In general, for SERS sequencing, a huge technical problem needs to be solved, i.e., the long integration time required to collect the spectra. As proposed here, ultrafast detection based on single photon detectors coupled with filters tuned to well separated wavenumbers can be explored as a potential solution to this limit.

To conclude, we want to briefly discuss potential future directions in the field of plasmonic nanopores. Two examples worthy to be mentioned, i.e., in situ single cells sequencing and DNA data storage. In situ single cells analysis is a promising direction for the application of plasmonic nanopores because the local DNA or protein sequencing can provide valuable biological information that no other techniques can acquire. To achieve this target, plasmonic nanopores combined with nanopipets or nanotubes in the patch clamp technique are feasible configurations to reduce possible interferences in the highly complexed intracellular environment. Nanopipets can insert into a cell easily without causing extra cell damage.^117^ Combining the plasmonic nanopore with theta or a multichannel nanopipet, one can deliver catabolic enzymes or denaturing reagents to decompose the chromosome or denature the protein in situ. Despite the many challenges, single molecule analysis or even single-molecule sequencing in the single cell is promising to be achieved using a SERS-plasmonic nanopore. The second example is in regards to the use of solid-state nanopores and plasmonic nanopores, in particular, to perform “sequencing” of modified DNA strands where binary information can be stored. DNA data storage is now an extremely interesting topic of research that is developed in parallel with single-molecule sequencing.^132−135^ In this field, solid-state nanopores are now used to retrieve the information stored in synthetic DNA,^132,136^ and it is possible to foresee the use of optical spectroscopies to improve the degree of freedom in the binary encoding, with a primary role of plasmonic nanopores in reading the information with electro-optical read-out methods.
